# Burkitt-type lymphoma incidentally found as the cause of acute appendicitis: a case report and review of literature

**DOI:** 10.1186/s40792-021-01283-8

**Published:** 2021-09-24

**Authors:** Davit Shahmanyan, Brian Saway, Hannah Palmerton, John S. Rudderow, Christopher M. Reed, Terri-Ann Wattsman, Emily R. Faulks, Bryan R. Collier, Robert E. Budin, Mark E. Hamill

**Affiliations:** 1 Department of Surgery, University of California San Francisco Fresno, 2823 Fresno St, Fresno, CA 93701 USA; 2 Department of Neurosurgery, Medical University of South Carolina, 96 Jonathan Lucas Street, Charleston, SC 29425 USA; 3 Department of Surgery, Madigan Army Medical Center, 9040A Jackson Ave, Joint Base Lewis-McChord, WA 98431 USA; 4 Department of Surgery, Virginia Tech Carilion School of Medicine and Research Institute, 3 Riverside Circle, Roanoke, VA 24016 USA; 5 Valley Health Bariatric and Metabolic Surgery Program, 1870 Amherst St, Winchester, VA 22601 USA; 6 Department of Surgery, University of Nebraska Medical Center, 983280 NE Medical Center, MSB 4553, Omaha, NE 68198-3280 USA; 7 Present Address: Virginia Tech Carilion School of Medicine and Research Institute, 3 Riverside Circle, Roanoke, VA 24016 USA

**Keywords:** Burkitt lymphoma, Acute appendicitis, Surgical pathology, Incidental finding

## Abstract

**Background:**

Appendectomy remains one of the most common emergency operations. Recent research supports the treatment of uncomplicated appendicitis with antibiotics alone. While nonoperative management of appendicitis may be safe in some patients, it may result in missed neoplasms. We present a case of acute appendicitis where the final pathology resulted in a diagnosis of a Burkitt-type lymphoma.

**Case presentation:**

An 18-year-old male presented to the emergency department with 24 h of right lower quadrant pain with associated urinary retention, anorexia, and malaise. Past medical history was significant for intermittent diarrhea and anal fissure. He exhibited focal right lower quadrant tenderness. Workup revealed leukocytosis and CT uncovered acute appendicitis with periappendiceal abscess and no appendicolith. Laparoscopic appendectomy was performed and found acute appendicitis with associated abscess abutting the rectum and bladder. Pathology of the resected appendix reported acute appendicitis with evidence of Burkitt-type lymphoma. A PET scan did not reveal any residual disease. Hematology/oncology was consulted and chemotherapy was initiated with an excellent response.

**Conclusions:**

Appendiceal lymphomas constitute less than 0.1% of gastrointestinal lymphomas. Primary appendix neoplasms are found in 0.5–1.0% of appendectomy specimens following acute appendicitis. In this case, appendectomy allowed for prompt identification and treatment of an aggressive, rapidly fatal lymphoma resulting in complete remission.

## Background

While acute appendicitis is a common pathological entity, it is a surgical emergency. The lifetime risk of developing appendicitis ranges from 7 to 8%, with the median age of diagnosis between 10 and 11 years of age [1]. The pathogenesis of acute appendicitis centers around luminal obstruction leading to inflammation, rising intraluminal pressure, and ultimately ischemia. Consequently, the appendix enlarges and leads to inflammatory changes in nearby tissue. While luminal obstruction is the ubiquitous primary event that sets off this inflammatory sequence of events, the cause of luminal obstruction includes fecalith, lymphoid hyperplasia, foreign bodies, and cancer [2]. While appendiceal lymphomas constitute 0.015% of gastrointestinal lymphoma cases, primary appendix neoplasms are found in 0.5–1.0% of appendectomy specimens following acute appendicitis [3].

Effective management of appendicitis is a topic of debate, with considerable options for operative and nonoperative approaches. Nonoperative management has become increasingly popular in recent years. A recent study demonstrated 4.9% of cases in 2014 were managed nonoperatively, which represents a 4.7% increase per year since 1998 [4]. While nonoperative management of acute uncomplicated appendicitis has a success rate of 75% at 1 year, we argue that operative management should not be hastily disregarded in this disease [5].

## Case presentation

Our patient was an 18-year-old male who presented to the emergency department with a complaint of approximately 24 h of pelvic pain. The symptoms were worsening and associated with urinary retention, nausea, and anorexia. The pain was exacerbated by movement. He reported similar symptoms approximately 1 month prior, with a negative workup by his primary care physician at that time. Past medical history was significant for intermittent loose stools, anal fissures, and a prior cyst in his neck. Surgical history included a colonoscopy as part of a workup for hematochezia several years earlier, which diagnosed an anal fissure. Excision of a reportedly benign neck cyst was also completed previously; however, no pathology was available.

**Fig. 1:**
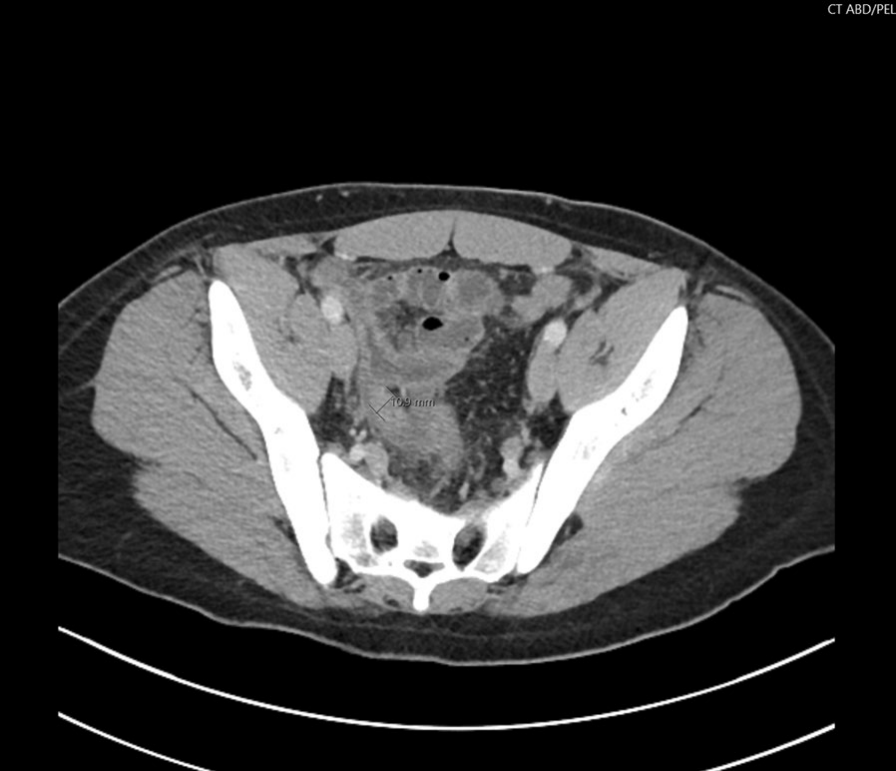
Transverse view of the abdominal CT demonstrating 0.9-mm dilated appendix with periappendiceal fluid in the pelvis

**Fig. 2:**
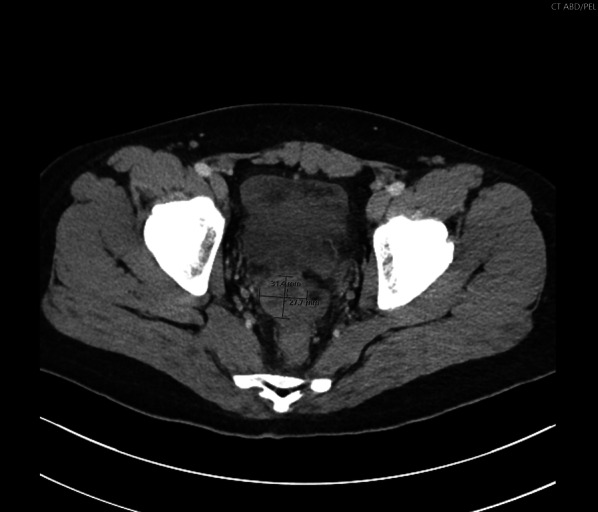
Transverse view of the abdominal CT demonstrating 31.4 × 27.7-cm appendiceal abscess by the appendiceal tip in the pelvis

On presentation, he was a well-appearing young adult male in mild distress. He was afebrile, and his vital signs were within normal limits, without evidence of tachycardia, tachypnea, or hypotension. His abdomen was soft but tender to palpation in the right lower quadrant, without evidence of generalized peritonitis. Lab values were insignificant except for leukocytosis (WBC 17.4 k) and mild hyperglycemia (blood glucose level 124 mg/dl). Computed tomography imaging obtained prior to the surgical consult demonstrated dilation of the appendix up to 11 mm with periappendiceal fluid (Fig. 1) and a 3-cm abscess adjacent to the appendiceal tip (Fig. 2) consistent with appendicitis with perforation and locally contained abscess.

Treatment options included appendectomy, treatment with antibiotics, and percutaneous drainage of the abscess, all of which were discussed with the patient and his family. After considering options, the patient and team elected to proceed to the operating room for laparoscopic appendectomy and drainage of the abscess. Antibiotic treatment with piperacillin/tazobactam (Zosyn) was initiated and the patient brought to the operating room and placed under general anesthesia for the procedure. Operative findings included purulent fluid throughout the peritoneal cavity with the appendix laying down in the pelvis. Appendiceal inflammation with obvious perforation to the appendiceal tip and adjacent abscess was noted. The patient’s postoperative course was significant for persistent nausea for 12 h after the procedure, which was treated with antiemetics and resolved. He was discharged to home on postoperative day #1 with a plan to complete a 10-day course of oral amoxicillin/clavulanic acid (Augmentin).

**Fig. 3:**
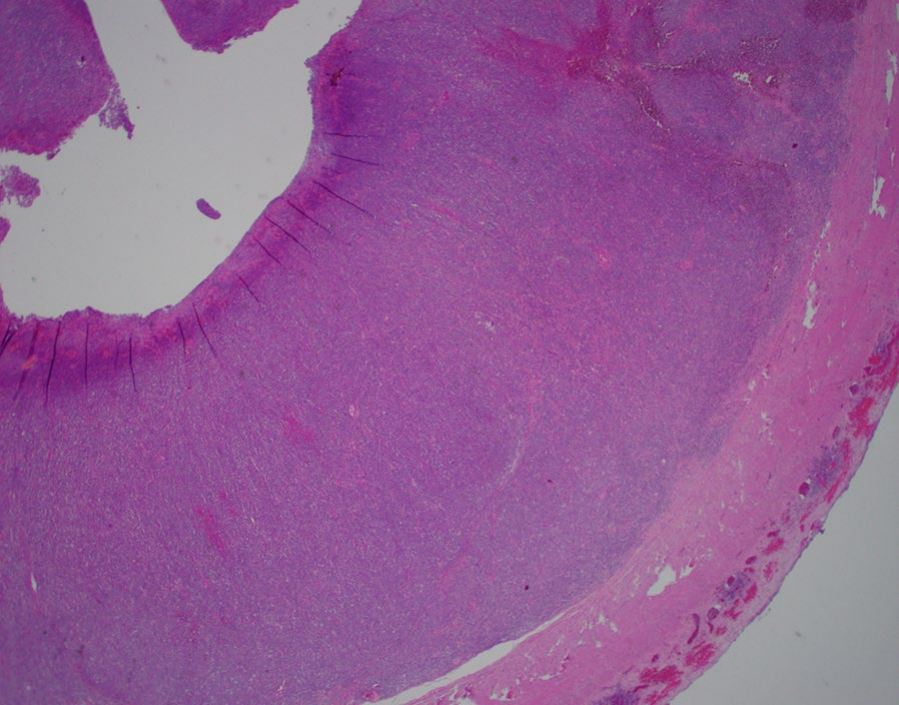
Hematoxylin and eosin staining of appendiceal tissue demonstrating significant lymphoid invasion (×20 magnification)

**Fig. 4:**
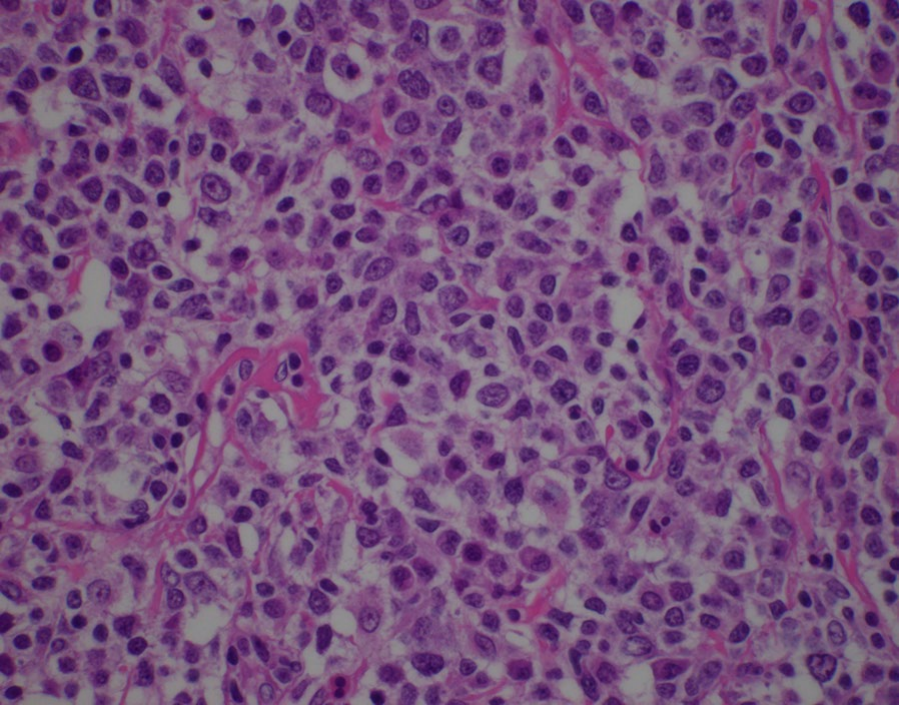
Hematoxylin and eosin staining of appendiceal tumor (×600 magnification)

**Fig. 5.**
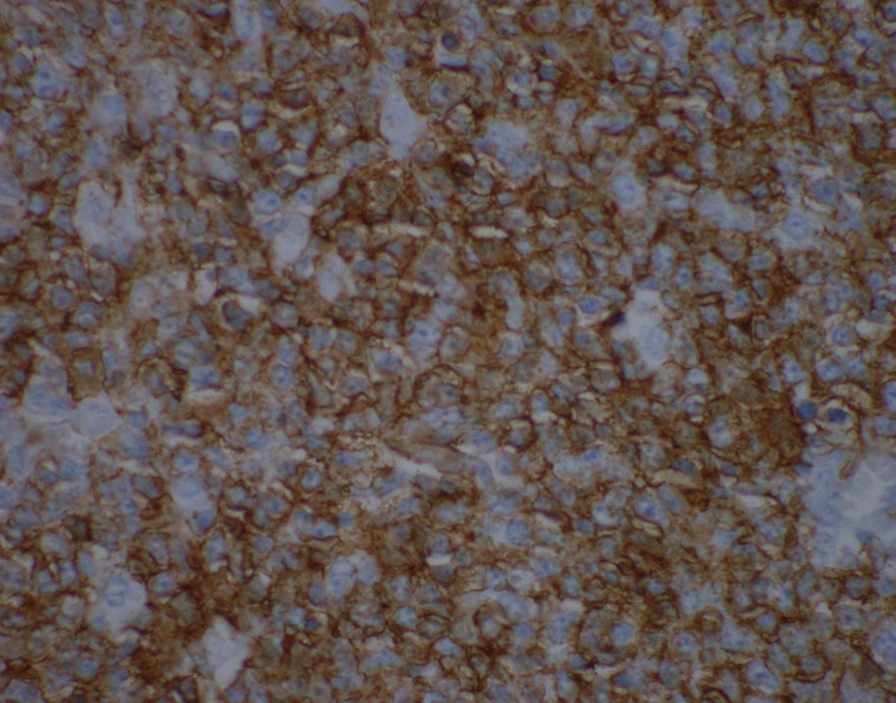
×600 Magnification of appendiceal tissue with CD20 immunohistochemistry staining

**Fig. 6.**
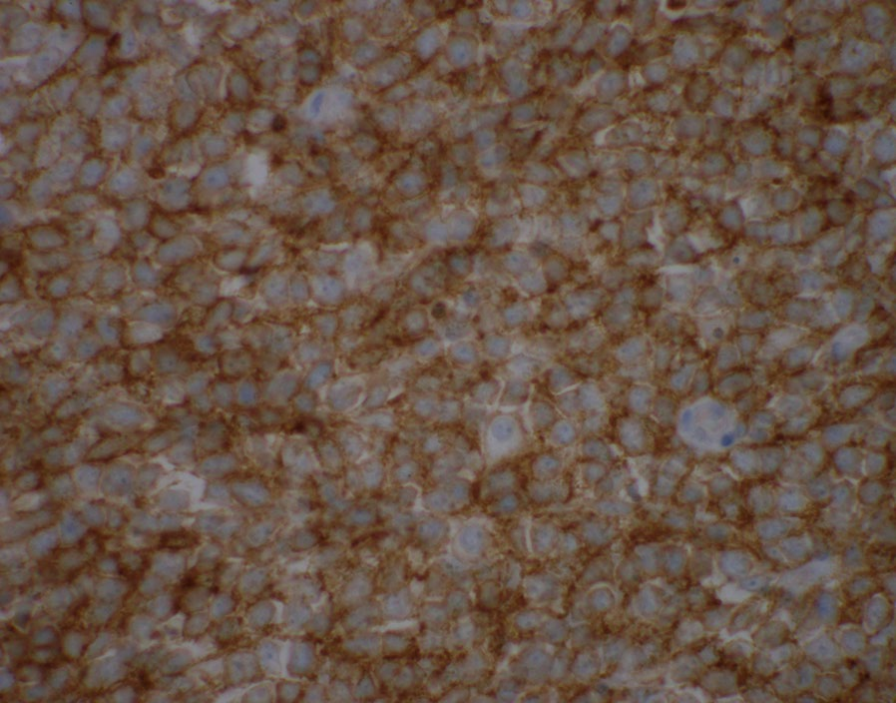
×600 Magnification of appendiceal tissue with CD10 immunohistochemistry staining

Several hours after his discharge, an urgent call was received from the pathologist reviewing the appendix specimen. Surprisingly, the appendix demonstrated a high grade transmural lymphoid malignancy with extra-appendiceal deposits (Figs. 3 and 4). The pathological diagnosis was initially classified as Burkitt lymphoma, which was later reclassified based on additional pathologic staining as a B-cell lymphoma with features between diffuse large B-cell lymphoma and Burkitt lymphoma. Positive tumor markers included CD20 (Fig. 5), CD10 (Fig. 6), BCL6, and Ki67. Urgent surgical follow-up and hematology/oncology consultation was obtained.

Given the Burkitt-type features and the potentially rapid progression of a Burkitt lymphoma, the decision was made to treat as a confirmed Burkitt lymphoma. The patient promptly underwent PET scanning, lumbar puncture, and port placement. PET imaging revealed no evidence of distant disease. He also had sperm banking performed to preserve the potential for future fertility. After sperm banking, the patient underwent a total of four cycles of cyclophosphamide, vincristine, doxorubicin, high-dose methotrexate, ifosfamide, etoposide, high-dose cytarabine (CODOX-M/IVAC) chemotherapy with an excellent response. Now more than 30 months out from treatment, he remains in complete remission.

## Discussion

The gastrointestinal tract is the most common extranodal sites of lymphoma metastases, accounting for 5–20% of all cases [6]. Primary GI lymphoma, however, is exceedingly rare and constitutes only about 1–4% of all GI malignancies as the majority occurs secondary to widespread nodal disease [7]. While this disease can manifest in any part of the GI tract, the most frequent sites of occurrence are the stomach, small intestine, and colorectal region. Of the three locations, the colorectal region, including the appendix, is the least common site constituting 6–12% of GI lymphomas [8]. While the clinical presentation and epidemiology of primary lymphoma of the colorectal region has been well studied, primary appendiceal lymphoma is a rare entity that is not yet clinically and prognostically well defined. The largest study to date assessed 116 patients with primary appendiceal lymphoma with a mean age at diagnosis of 48 years [3]. The study also found that males and Caucasians were significantly more affected, and diffuse large B-cell lymphoma was the most common histologic subtype (34.5%). Burkitt lymphoma is the second most common cause of appendiceal lymphoma. It is a subtype of non-Hodgkin lymphoma that is highly aggressive and rapidly fatal if left untreated. Patients with Burkitt lymphoma generally do not present with classic B-symptoms suggestive of malignancy, making it commonly found later in its clinical course, with subsequently poor outcomes [9]. Additionally, in Burkitt lymphoma of sporadic origin, presenting abdominal symptoms may mimic that of acute appendicitis. When the lymphoma presents within the appendix, the resulting acute appendicitis and appendectomy with histological analysis provide clinicians with an early diagnosis and an opportunity for early treatment [4, 10, 11].

Interestingly, patients with Burkitt lymphoma of the appendix presented at an earlier age (33 years) compared with follicular lymphoma (59 years) and diffuse large B-cell lymphoma (53 years), which is consistent with our case involving a young white male patient. The mortality rate of primary appendiceal lymphoma is promising, as the 5-year survival rate was found to be 67% in the cohort studied. Similarly, the 5-year survivor rate of Burkitt lymphoma/leukemia is 87% in ages 0–19 and 60% in those 20–39 [12]. The clinical presentation widely varies as the patients can present with symptoms of intestinal obstruction and GI bleeding, or it can manifest as acute appendicitis as the expansion of tissue leads to obstruction and subsequent inflammation and rupture of the appendix. For primary appendiceal lymphoma, early diagnosis and definitive treatment are essential as delay in treatment can lead to local and lymphatic spread of the disease, leading to increased mortality. In this case, there was some question of the final pathologic diagnosis as the lymphoma had staining characteristics which were somewhat atypical. This is not uncommon and has led to a reclassification of these tumor types [13]. However, due to the aggressive and fatal nature of untreated Burkitt lymphoma the decision was made by the oncologists to proceed with rapid and aggressive treatment assuming typical Burkitt lymphoma features were present.

CT Imaging is a vital tool for diagnosis of appendiceal neoplasms by demonstrating the disproportionately increased diameter of the appendix shown on CT-scans (> 3 cm). In contrast, non-tumoral appendicitis does not have as large a diameter. CT Imaging is also critical in assessing the extent of tumor burden throughout the body [14]. However, in the case of primary appendiceal lymphoma presenting as acute appendicitis, the decision to utilize CT is still controversial. While CT-scanning grants over 90% sensitivity and specificity for acute appendicitis and helps with other differential diagnoses, it is not mandatory as a diagnosis of acute appendicitis can be made clinically [8].

## Discussion regarding medical decision-making we made during the case

Timely appendectomy, either open or laparoscopic, has been the recommended treatment of choice for acute appendicitis due to recent advances in perioperative management, which has lowered rates of wound infection, shortened hospital stays, and significantly decreased morbidity [15]. However, recent research has provided evidence for nonoperative management of acute appendicitis. To date, six randomized trials have compared antibiotics with appendectomy for nonperforated appendicitis in adults, reporting a reduction in leukocytosis, avoidance of peritonitis, and general symptom reduction with antibiotic treatment alone. Furthermore, these studies state that 90% of patients treated with antibiotics can avoid surgery during the initial admission, and 70% of those successfully treated with antibiotics during the initial admission can avoid surgery during the 1st year [5, 16–20]. Unfortunately, only one study analyzed follow-up data beyond the 1st year of treatment of acute appendicitis with antibiotics and found a 39.1% incidence of recurrent appendicitis at 5 years [21]. The Comparison of Outcomes of antibiotic Drugs and Appendectomy (CODA) randomized controlled study, which is ongoing, aims to examine if antibiotic therapy is non-inferior to surgery, and will have follow-up of 2 years to see if any harm results from nonoperative therapy [22]. While this research provides a compelling argument for consideration of antibiotics alone for treatment of acute appendicitis, there is more recent literature which speaks to the concern of whether treatment with antibiotics alone can lead to missed or delay in diagnoses of primary neoplasms. This would have been the case in our report. For adults the risk of an appendiceal cancer is not insignificant in those presenting with appendicitis. Lu et al. found when reviewing National Surgical Quality Improvement Program (NSQIP) data, that in a cohort of 21,069 patients from 2016 to 2017 who underwent appendectomy for either imaging-proven appendicitis or equivocal for appendicitis imaging, that the odds ratio of being diagnosed with cancer increased with each decade after age 50 up to age 80 [23]_._ Skendelas et al. found a 0.53% incidence of appendiceal malignancies in NSQIP database patients who had undergone appendectomy for appendicitis from 2010 to 2018, and 1.7% incidence in their local patients who had surgery at two hospitals in The Bronx, New York City [24]. Finally, the recent Peri-Appendicitis Acuta randomized trial looking at the necessity of interval appendectomy after successful nonoperative treatment of peri-appendicular abscess was terminated early basic on ethical concerns after a neoplasm rate of 20% was discovered in patients who underwent interval appendectomy [25].

## Conclusion

While new research has provided evidence for antibiotics alone as treatment for acute appendicitis, we believe it will lead to missed or delayed diagnoses of primary appendix neoplasms. Our patient discussed above presented with acute appendicitis, he was treated with appendectomy and was diagnosed with Burkitt-type lymphoma of the appendix on pathology. Due to the early diagnosis, this patient was able to be rapidly and effectively treated resulting in a complete remission and a significant improvement in his prognosis. Had he been treated with antibiotics alone, even if it was successful in the control of his appendicitis, the chances of a poor outcome from a later diagnosis of Burkitt lymphoma is very high. This potential risk needs to be considered when examining the risks and benefits of operative versus nonoperative management of acute appendicitis.

## Data Availability

All data generated or analyzed during this study are included in this published article.

## Abbreviations

CT: Computed tomography
PET: Positron emission tomography
CODOX-M/IVAC: Cyclophosphamide, vincristine, doxorubicin, high-dose methotrexate/ifosfamide, etoposide, high-dose cytarabine
GI: Gastrointestinal
WBC: White blood cell
